# rs12480307 and rs6050307 Polymorphisms of VSX1 Gene in Patient with Keratoconusin Southwest Iran Using PCR-RFLP

**Published:** 2018-03

**Authors:** Raziyeh KARAMI ESHKAFTAKI, Effat FARROKHI, Fatemeh HEYBATI GOJANI, Najmeh SALEHI VANANI, Maryam KARAMI ESHKAFTAKI, Ezzatollah MEMARZADEH, Morteza HASHEMZADEH CHALESHTORI

**Affiliations:** Cellular and Molecular Research Center, Basic Health Sciences Institute, Shahrekord University of Medical Sciences, Shahrekord, Iran

## Dear Editor-in-Chief

Keratoconus (KC) is disorder of the eye characterized by progressive thinning of the cornea. Several genes have been associated with KC disease. Both genetic and environmental factors are associated with KC (1, 2).

KC is one of the leading indicators for corneal transplantation in the Western countries (3, 4). KC usually occurs in the second decade of life (5, 6). Although the disease has been reported at an earlier age and adolescence (7) and classically progresses until the third or fourth decade of life (6). Incidence disease is estimated between 1500 to 12000 people worldwide (6). KC prevalence in first degree relatives is 15 to 67 times more than that in the general population (8). Both autosomal dominant and recessive forms of inheritance have been determined in KC pedigrees, but can be seen in over 90% of autosomal dominant inheritance with reduced expression (9, 10). Despite extensive studies, the pathophysiology processes and genetic etiology of KC are still unknown (1).

This study aimed at investigating the prevalence of genotypic and allelic single nucleotide polymorphisms (SNPs) in *VSX1* gene and its relationship with KC disease in Iranian population by PCR-RFLP method.

In total, 100 healthy control (36 males, 64 females) subjects and 100 patients (53 males, 47 females) with KC were enrolled. The study population included all patients with KC who were referred to Kashani Hospital, Chaharmahal va Bakhtiari Province, Southwest of Iran, between September 2015 and March 2016. Diagnosis of KC was based on clinical examinations and the presence of characteristic topographic features.

Written informed consent forms were obtained from all participants. This research study was approved by the Ethics Committee of the Shahrekord University of Medical Sciences.

**Fig. 1:**
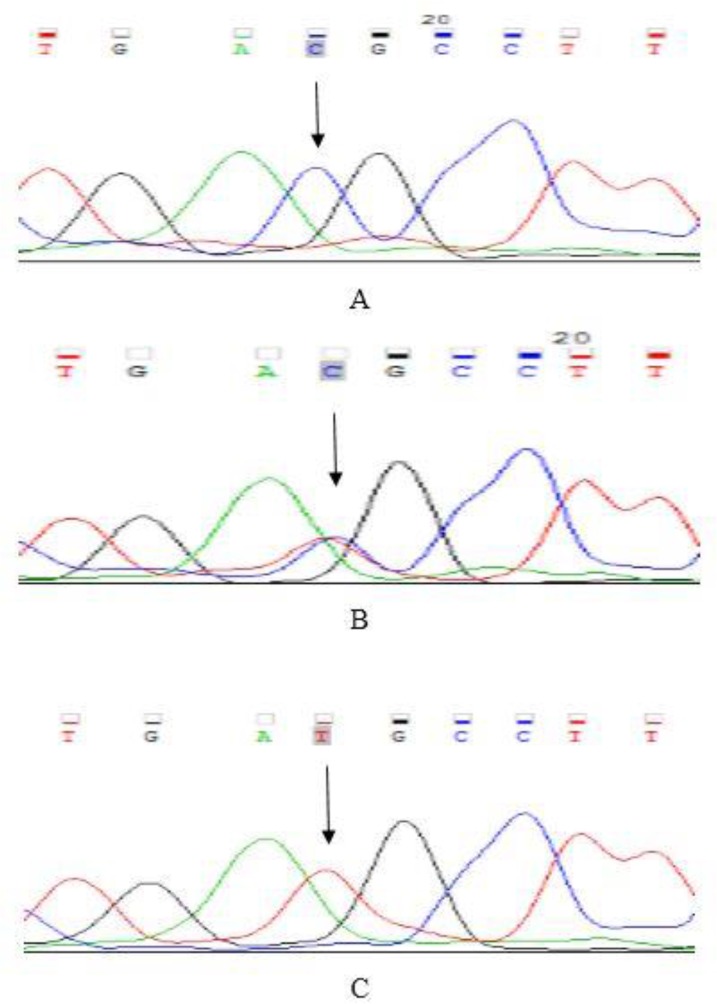
Electropherogram of rs12480307 polymorphism in *VSX1* gene A: Homozygous CC. B: Heterozygous CT. C: Homozygous TT

Statistical analysis was performed using the SPSS 16 (Chicago, IL, USA). Chi-square test was used to compare the genotypes and *P*<0.05 was considered statistically significant. Sequencing was performed by Macrogen South Korea (Fig. 1).

We found a significant association between the rs12480307 polymorphism of the *VSX1* gene and KC risk (*P*<0.05). The frequency of heterozygous genotype in patients (52%), in comparison with control subjects (31%), indicate that the polymorphism rs12480307 may play a role in KC. An adenine to guanine substitution occurs in coding region of exon3 of *VSX1* gene in rs12480307.

Change at codon 182 amino acid position, (GCA> GCG) and therefore synonymous change of p.Ala182Ala is highly conserved throughout many species. The allelic frequency rs12480307 polymorphism *VSX1* gene showed no significant association between two groups of patients and controls.

We observed no association between the rs6050307 polymorphism of the *VSX1* gene and the risk of KC. We compared the genotype and allele frequency polymorphism between the patients and healthy controls. In rs6050307 polymorphism a cytosine nucleotide replaces with adenine nucleotide at codon 131 (CGC> AGC) and therefore non-synonymous change in p.Arg131Cys occurs. Separate analyses in males and females subjects did not reveal sex-related associations of specific genotypes or alleles frequency of the two polymorphisms with KC. The observed genotypes frequencies of rs6050307 polymorphism did not deviate statistically significantly from those expected from the Hardy–Weinberg equilibrium (Table 1).

**Table 1:** Frequencies of *VSX1* gene variants in KTCN cases and healthy control

***VSX1 gene variations***			***Case***		***Control***		***P***	***OR***	***CI(95)%***

			**n=100**	**%**	**n=100**	**%**			
C.546A>G (rs12480307)	Genotype	CC	10	10	13	13	0.79	1.13	0.45–2.85
		CT	52	52	31	31	0.003	2.47	1.35–4.53
		TT	38	38	56	56			
	Allele	C	72	36	57	28.5	0.109	1.41	0.926–2.151
		T	128	64	143	71.5			
C.426C>A (rs6050307)	Genotype	GG	91	91	88	88	0.489	1.38	0.55–3.4
		GT	9	9	12	12			
	Allele	G	191	95.5	188	94	0.501	1.355	0.558–3.29
		T	9	4.5	12	6			

OR-odds ratio, CI- confidence interval, p-value less than 0.05 was considered as significant

We have assessed the role of *VSX1* by PCRRFLP in 2 polymorphisms in 100 patients KC and 100 healthy controls. Altogether, rs12480307 of VSX1 gene maybe involved as a risk factor in the pathogenesis of KC.
